# Prognostic value of non-specific ST-T changes and left ventricular hypertrophy electrocardiographic criteria in hypertensive patients: 16-year follow-up results from the MINACOR cohort

**DOI:** 10.1186/s12872-015-0012-6

**Published:** 2015-03-18

**Authors:** Ernest Vinyoles, Núria Soldevila, Joan Torras, Noemí Olona, Mariano de la Figuera

**Affiliations:** La Mina Primary Care Center, University of Barcelona, Barcelona, Spain; Barcelona Primary Care Catchment Area, Catalan Health Institute, Barcelona, Spain; Sardenya Primary Care Center, Barcelona, Spain; CAP La Mina, Carrer Mar s/n, 08930, Sant Adrià de Besòs, Barcelona, Spain

**Keywords:** Electrocardiogram, Cardiovascular events, Hypertension, Left ventricular hypertrophy, Major and minor electrocardiographic abnormalities, Repolarization electrocardiographic abnormalities

## Abstract

**Background:**

Non-specific electrocardiographic ST-T wave changes and voltage criteria for left ventricular hypertrophy (LVH) have been associated with cardiovascular morbidity and mortality. The aim of the cohort study was to evaluate the prognostic value of non-specific ST-T changes and LVH electrocardiographic criteria on cardiovascular events and mortality in hypertensive patients.

**Methods:**

A cohort study of 352 non-diabetic hypertensive patients, without associated cardiovascular disease, randomly selected from 1,780 hypertensive patients attended in a primary care center. An electrocardiogram was performed at the baseline visit (classified according to the Minnesota Code). Cardiovascular events and death from any cause during the follow-up period were evaluated. A multivariate analysis adjusted for gender, age and cardiovascular risk factors was performed.

**Results:**

Data of 273 patients were analyzed: 58.2% women, age 44.1 (7.9) years, 27.8% smokers, blood pressure at baseline 142.7 (15.3)/89.3 (9.6) mmHg. During the 197.5 (59.24) month follow-up, 62 patients (22.7%) had a cardiovascular event. On multivariate analysis, age, systolic blood pressure, incidence of diabetes, smoking and electrocardiographic LVH criteria (HR 2.66 [CI 95% 1.39 – 5.10]), were significantly associated with cardiovascular events, but the presence of non-specific ST-T abnormalities (HR 0.97 [CI 95% 0.49 -1.90]) was not significantly associated with cardiovascular morbidity and mortality.

**Conclusions:**

Hypertensive patients with LVH electrocardiographic criteria have significantly higher cardiovascular mortality and morbidity, but non-specific electrocardiographic ST-T changes are not associated with cardiovascular morbidity and mortality.

## Background

The electrocardiogram (ECG) is a routine, accessible, cost-effective and recommended diagnostic tool for the initial evaluation and follow-up of hypertensive patients. Despite its limitations, the utility of this exam cannot be denied, both for the detection of left ventricular hypertrophy (LVH) and for cardiovascular risk stratification and assessment of arrhythmias, blockages or coronary artery disease [1].

Non-specific repolarization disorders represent the most prevalent electrocardiographic abnormalities in hypertensive patients [2] with an uncertain prognostic significance. Many studies significantly associate non-specific ST-T segment abnormalities with cardiovascular morbidity and mortality, regardless of the populations or ethnic origin of the patients [3-12]. In contrast, other studies found no significant association during follow-up, at least in some subgroups of patients [13-15]. This prognostic variability is probably related to the differences in the design, particularly regarding the follow-up time, the number of patients included in each study, and the different inclusion criteria used. Therefore, our study aims to provide information in this regard from a cohort of hypertensive patients treated within a primary care setting.

The goal of our study was to determine whether non-specific baseline ST-T ECG abnormalities and LVH voltage criteria during follow-up were associated with a higher incidence of cardiovascular events in hypertensive patients.

## Methods

This is a prospective cohort study (MINACOR) that included 352 hypertensive, non-diabetic patients with no associated cardiovascular disease, registered and treated at *La Mina Primary Care Center*, who were followed-up from January 1992 to September 2011. *La Mina Primary Care Center* is located in Sant Adrià de Besòs, municipality of Barcelona, and comprises 11 primary care offices. At present, our primary care center provides medical services to an urban catchment area of 11,373 adult patients, of which 26.73% are hypertensive (according the our electronic medical records data base).

The design, structure and ethical points of the study were approved by the Ethics Commission of Teaching Unit of Family and Community Medicine in Barcelona in 1991. All patient information was treated confidentially in accordance with applicable law in Catalonia, with standards of good clinical practice and in compliance with the Helsinki Declaration. Informed consent was obtained from patients and it was a compulsory inclusion criteria.

The MINACOR cohort was started in 1992 with a baseline visit. Over time, this cohort of patients has provided information on the incidence of electrocardiographic abnormalities in hypertensive patients and on predictor variables of electrocardiographic changes in the first few years of follow-up [16-19].

Patient selection was made by systematic random sampling over a total of 1,780 hypertensive patients <55 years, registered and attended at the center in 1992. The criteria for hypertension that were in place at that time were used: mean blood pressure of three measurements at three different visits ≥140/90 mmHg.

Patients with diabetes mellitus, or a prior diagnosis of coronary artery disease, heart failure, valvular disease, or atrial fibrillation were excluded [17].

Included patients had and ECG at the baseline visit that was interpreted and classified according to the Minnesota Code [20]. The design was previously published [19].

The baseline visit included the collection of demographic data, office blood pressure values (average of two seated readings using a calibrated mercury sphygmomanometer, at a one minute interval from each other, and a special cuff for obese patients, when necessary), obesity (defined by a body mass index ≥ 30 kg/m^2^), hypercholesterolemia (total cholesterol >250 mg/dl or LDL cholesterol >155 mg/dl, or HDL cholesterol <40 mg/dl in men or <48 mg/dl in women, or the use of lipid-lowering medications [21], diagnosis of active smoking (defined as a daily consumption of any type of tobacco), heavy alcohol consumption (>40 g/day for women and >80 g/day for men), presence of electrocardiographic abnormalities and antihypertensive drug treatment.

All ECGs were read only by two trained professionals from the center (EV and MF). When there was disagreement between the two readers regarding the interpretation of the ECG, a third reading was done jointly with the aim of reaching a final interpretation of the ECG. The Cornell index (for men: RaVL + SV_3_ > 2.8 mV; for women: RaVL + SV_3_ >2.0 mV) and the Sokolow-Lyon index (SV_1_ + RV_5_ or V_6_> 3.5 mV) were evaluated as electrocardiographic LVH criteria.

The incidence of diabetes mellitus (defined as 2 or more basal glycemia ≥ 126 mg/dl) [22], the incidence of cardiovascular disease [cerebrovascular disease (stroke or transient ischemic attack); dementia from all causes; coronary heart disease (myocardial infarction, stable angina or myocardial revascularizations); heart failure; chronic kidney disease (defined by the estimated glomerular filtration rate according to the Cockroft-Gault formula normalized per 1.73 m^2^ <60 ml/min) [23] or peripheral arterial disease (intermittent claudication, ankle brachial index< 0.9 or carotid stenosis)], according to the patient’s clinical records throughout the follow-up period, were assessed during the entire course of the follow-up period.

The primary endpoint was a composite of fatal or non-fatal cardiovascular events. In addition, death from any cause during the follow-up period was also recorded. The detection of cardiovascular events was conducted using three methods: (1) review of the shared electronic medical record for use in the primary care system of the Catalan Institute of Health (ECAP), which includes 5.8 million people attended in Catalonia; (2) a review of the medical records in a paper format used at our center (records between 1984 and 2005); and, (3) the mortality registry of our center (updated monthly since 1986). When, despite these three ways, the information could not be completed, each patient’s GP was asked directly at the actual center (most GPs have been seeing patients continuously at the center well before 1992 and the migration pattern of the population attended is small). The doctors of those patients whom we had been unable to reach contacted their families to obtain the information.

Electrocardiographic abnormalities were divided into major and minor abnormalities according to the Minnesota Code (MC). Criteria for major ST-T abnormalities were: MC 4–1, 5–1, and 5–2. Criteria for minor ST-T changes were: MC 4–3 and 5–3. Patients with major and minor ST-T abnormalities were considered and analyzed as having major ST-T changes.

### Statistical analysis

Incidence rates per 1000 patients/year were calculated for both cardiovascular morbidity and mortality (having suffered a cardiovascular event or death from cardiovascular causes) and for each cardiovascular event and mortality from cardiovascular causes.

The 95% confidence intervals were calculated assuming a Poisson distribution.

Survival curves were calculated using the Kaplan-Meier method for cardiovascular morbidity and mortality separately for those who had or did not have any non-specific ST-T abnormalities, major ST-T abnormalities, minor ST- T and LVH according to electrocardiographic criteria. The comparisons between survival curves, according to independent variables, were calculated using the Log-Rank test.

Cox proportional hazards models were estimated to assess the relationship between variables and the occurrence of events (cardiovascular morbidity and mortality) adjusted for the follow-up time. Marginally significant variables, those considered clinically relevant, and all major interactions were included in the univariate analysis (p <0.10). The hazard ratio (HR) estimation and its 95% confidence interval are provided for each of the final models.

A combined variable was defined as the primary endpoint of the study: time free of cardiovascular disease or death from a cardiovascular origin (cardiovascular morbidity and mortality).

## Results

A total of 352 patients were initially included, of which 77 were lost to follow-up. Two other patients were withdrawn from the study because they had an illegible ECG at baseline. Finally, 273 hypertensive patients were assessed. The majority of them were women (58.2%) with a mean age of 44.1 (7.9) years. From the total number of patients, nearly one third were smokers. At the baseline visit, the mean values of systolic and diastolic blood pressure were 142.7 (15.3) mmHg and 89.3 (9.6) mmHg, respectively. A total of 173 patients were under antihypertensive treatment (121 patients with one drug, 43 with two drugs and 9 with three drugs), and 19 (7%) patients had subclinical organ damage at the baseline visit, according to medical records. Nevertheless the prevalence of LVH was 8.7% (Cornell criteria). No patient met Sokolow-Lyon criteria.

At the baseline visit, 52 patients had some sort of ST-T electrocardiographic abnormality or Cornell criteria: 17 patients had isolated LVH criteria, 28 patients had an isolated ST-T change (16 patients with major abnormalities and 12 patients with minor abnormalities), and 7 patients had both LVH and ST-T abnormalities (3 patients with major abnormalities and 4 patients with minor abnormalities) (Table 1).There were no statistically significant differences between patients with and without electrocardiographic abnormalities, except for systolic blood pressure which was higher in subjects with electrocardiographic abnormalities [147 (15.8) mmHg vs. 141 (15.0) mmHg, p=0.03].

**Table 1:** **Baseline sociodemographic and clinical characteristics of the total sample (n=273)**

Women [n (%)]	160 (58.2)
Age [mean (SD)] (years)	44.1 (7.9)
Smoking [n (%)]	76 (27.8)
Heavy alcohol consumption [n (%)]	39 (14.3)
Blood pressure [mean (SD)] (mmHg)	
Systolic	142.7 (15.3)
Diastolic	89.3 (9.6)
Obesity [n (%)] (BMI ≥ 30 kg/m2)	168 (61.5)
Dyslipidemia [n (%)]	127 (46.5)
Patients with antihypertensive treatment [n (%)]	173 (63.3)
Diuretics	80 (29.1)
Beta blockers	54 (19.6)
Angiotensin-converting-enzyme inhibitors	54 (19.6)
Calcium channel antagonists	46 (16.7)
Baseline electrocardiographic ST-T changes and left ventricular hypertrophy* [n (%)]:	
Major ST (MC 9–2; 4–1; 4–2)	15 (5.5)
Inverse Major T wave (MC 5–1; 5–2)	6 (2.2)
Inverse Minor T wave (MC 5–3)	16 (5.9)
Minor ST (MC 4–3)	5 (1.8)
Left ventricular hypertrophy (Cornell criteria)	24 (8.7)

SD: standard deviation; BMI: body mass index; A-V: atrioventricular; MC: Minessota code.

*A total of 52 patients had 66 basal electrocardiographic abnormalities.

The mean follow of the sample was 197.5 months (59.24). The median follow-up among men was 205.7 months (95% CI 195.6 to 215.8) and 210.9 months among women (95% CI 203.5 to 218.4).

A total of 84 patients (30.5%) had incident diabetes mellitus during the follow-up time.

Of the 273 patients, 62 patients had 82 cardiovascular events during follow-up: 47 patients had one single cardiovascular event, 10 patients had two and 5 patients had three cardiovascular events (Table 2). During the follow-up period, 36 patients died (13.1%), 10 from cardiovascular causes (three patients died from cerebrovascular disease, three patients died from sudden cardiac death, two patients died from acute myocardial infarction and two patients died from chronic kidney disease).

**Table 2:** All cardiovascular events during follow-up (n=62 patients; 82 events*)

	***N***	***%***	**Incidence rate per 1000 patients/year [95% CI]**
Coronary heart disease	21(5^†^)	7.7	4.84 [3 – 7.4]
Peripheral artery disease	14	5.1	3.23 [1.8 – 5.4]
Chronic kidney disease	18 (2^†^)	6.6	4.15 [2.5 – 6.6]
Cerebrovascular disease	9 (3^†^)	3.3	2.07 [0.9 – 3.9]
Dementia	6	2.2	1.38 [0.5 – 3.01]
Heart failure	6	2.2	1.38 [0.5 – 3.01]
Other (not included in the primary endpoint)^	8	2.9	1.84 [0.8 – 3.6]

^*^47 patients had one cardiovascular event, 10 patients had 2 cardiovascular events and 5 patients had 3 cardiovascular events. Only first cardiovascular event was considered for the analysis.

^†^Number of deceased patients; ^ Atrial fibrillation (6 patients), Mobitz Type II A-V block (1 patient), subclavian artery stenosis (1 patient); CI: confidence interval.

**Figure 1:**
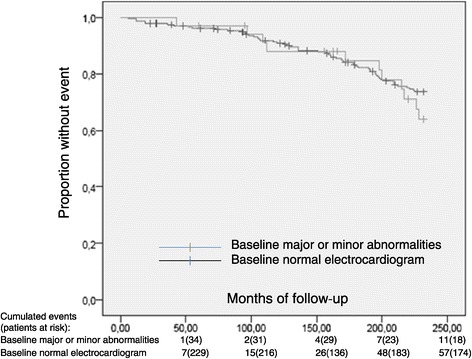
**Time to First Cardiovascular Event Kaplan-Meier Estimates (p=0.385).**

**Figure 2:**
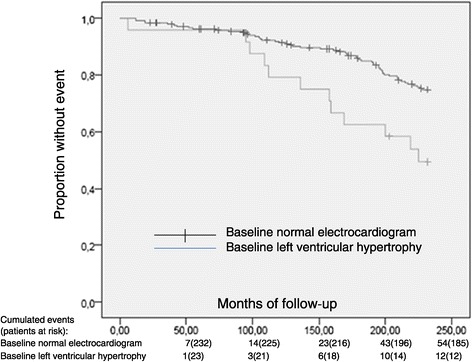
**Time to First Cardiovascular Event Kaplan-Meier Estimates (p=0.003) Months.**

With regard to the presence or absence of major or minor abnormalities as per the findings from the baseline ECG, there was no statistically significant difference in cardiovascular event-free survival [209.4 (8.0) vs 208.7 (3.3) months, p = 0.385 respectively)]. Isolated major or isolated minor ST-T changes didn’t predict cardiovascular events (the combined morbidity and mortality endpoint). However, a statistically significant difference was seen in event-free survival when patients with baseline electrocardiographic LVH criteria were compared to the rest of patients [186.1 (12.6) vs 211.0 (3.1) months, p = 0.003, respectively)] (Figure 1 and Figure 2).

Table 3 and Table 4 describes the variables associated with the incidence of cardiovascular events during the follow-up time. LVH was the only electrocardiographic abnormality that was significantly associated with cardiovascular endpoint (morbidity and mortality).

**Table 3:** **Risk for cardiovascular outcomes or cardiovascular mortality**

	**HR [95% CI] adjusted for gender, age, SBP, DM2 incidence, LVH and smoking**	***P*** **-value**	**Crude HR [95% CI]**	***P*** **- value**
LVH, Cornell criteria	2.66 [1.39 – 5.10]	0.003	2.47 [1.39 – 4.60]	0.005
Gender (Male)	0.58 [0.24 – 1.40]	0.223	1.18 [0.73 – 1.90]	0.491
Age	1.07 [1.03 – 1.12]	0.002	1.07 [1.02 – 1.11]	0.003
SBP	1.01 [0.99 – 1.03]	0.108	1.02 [1.00 – 1.03]	0.022
DM incidence	1.63 [0.99 – 2.68]	0.055	2.08 [1.30 - 3.30]	0.003
Smoking	0.68 [0.16 – 2.88]	0.603	1.71 [1.05 - 2.80]	0.033
Male * Smoking	6.17 [1.11 – 34.24]	0.038	2.00 [1.21 - 3.31]	0.007

Hazard ratios were estimated by using proportional hazards (Cox) regression modeling with LVH.

**Table 4:** **Risk for cardiovascular outcomes or cardiovascular mortality**

	**HR [95% CI] adjusted for gender, age, SBP, DM2 incidence, major or minor ECG abnormalities and smoking**	***P*** **-value**	**Crude HR [95% CI]**	***P*** **-value**
Major or minor ECG abnormalities	0.97 [0.49 -1.90]	0.923	1.33 [0.69 - 2.54]	0.387
Gender (Male)	1.08 [0.59 - 1.96]	0.804	1.18 [0.73 - 1.90]	0.491
Age	1.07 [1.03 - 1.12]	0.002	1.07 [1.02 - 1.11]	0.003
SBP	1.01 [1.00 - 1.03]	0.053	1.02 [1.00 - 1.03]	0.022
DM incidence	1.97 [1.21 - 3.20]	0.006	2.08 [1.30 - 3.30]	0.003
Smoking	1.98 [1.06 - 3.70]	0.032	1.71 [1.05 - 2.80]	0.033

LVH, Left Ventricular Hypertrophy; HR: Hazard Ratio; SBP, Systolic Blood Pressure; DM: Type 2 Diabetis; CI, confidence interval.

Hazard ratios were estimated using proportional hazards (Cox) regression modeling with major or minor electrocardiographic (ECG) abnormalities.

A separate subanalysis of cardiovascular morbidity and mortality was also performed. The hazard ratio (HR) for cardiovascular morbidity in patients with baseline LVH was 3.11 [CI 95% 1.61 – 6.01], p<0.001, and in patients with non-specific ST-T abnormalities was 0.88 [CI 95% 0.41 – 1.87], p=0.745, adjusting for age, sex, blood pressure, smoking and diabetes. When only cardiovascular mortality (n=10) was analyzed, LVH and the combined non-specific ST-T changes lost their predictive value. Nevertheless isolated major ST-T changes predicted cardiovascular mortality: HR 5.06 [CI 95% 1.20 – 21.36], p=0.027, adjusting for age, sex, smoking and heavy alcohol consumption.

## Discussion

Unlike other studies, our study does not demonstrate that non-specific abnormalities of the terminal segment of the electrocardiogram are associated with cardiovascular morbidity and mortality over a mean follow-up period of more than 16 years of a cohort of hypertensive patients. In contrast, statistically significant associations were indeed found with other previously known and assessed cardiovascular risk factors such as age, smoking (especially in males), the incidence of diabetes mellitus, systolic blood pressure and electrocardiographic LVH voltage criteria. These associations between classic risk factors and cardiovascular events are consistent with previous evidence. In a way, these associations confirm that patient follow-up quality and data collection is adequate. Hence, it indirectly reinforces the main finding yielded by our study, which is the lack of association between global major and minor ST-T segment changes and cardiovascular events in hypertensive patients. As discussed in the introduction section, other studies have also reached a similar conclusion. In the study by De Bacquer et al. [13] only certain non-specific changes of the terminal segment were associated with cardiovascular morbimortality, such as major abnormalities of the ST-T segment and, in contrast, this association did not occur with minor changes of the ST-T segment. In our study, when a separate mortality and morbidity sub analysis was performed, we also found a significant association between isolated major ST-T changes and cardiovascular mortality. Nevertheless the low number of cardiovascular deaths (n=10) does not allow to reach a definitive conclusion.

In other studies, such as the one by Liao et al. [14], there was only statistical significance for men -with increased cardiovascular risk- but not for women. It is possible that we may not have found any prognostic value of non-specific changes of the terminal segment due to the low baseline cardiovascular risk of the cohort. Although more than half of the sample consisted of obese and dyslipemic patients, in fact, these patients were hypertensive relatively young, with a mean age of 44 years, with only 7% of asymptomatic organ damage and without diabetes. Therefore, at the end of follow-up, most patients were about 60 years old. We do not know whether a longer follow-up, or older patients, would have been useful to demonstrate the global predictive value of non-specific abnormalities of ST-T segment. Instead, we found association between cardiovascular events and LVH, which is significant after 8 years of follow-up. Probably, ECG-detected LVH, poses a situation of increased cardiovascular risk than non-specific changes in the ST-T segment, although the later represent the most prevalent electrocardiographic abnormality shown in ECGs of hypertensive patients. Some years back, in that same cohort, it was demonstrated that the incidence of major or minor non-specific changes were associated to a worse control of blood pressure [17]. In fact, we found that patients with baseline electrocardiographic abnormalities had significantly higher systolic blood pressure than patients with a normal electrocardiogram. Therefore, non-specific ST-T changes could be early indicators of a poor management of blood pressure and, in the long term, could predict subclinical organ damage such as LVH itself. In any case, the design of our study cannot prove it and this approach is just a mere hypothesis that should be confirmed in other studies.

One of the limitations encountered is the lack of reproducibility of ST-T segment changes on successive ECG, and what the variability attributable to the observer is, especially in the case of minor abnormalities. We know that in terms of routine clinical practice, the reproducibility of Sokolow and Cornell electrocardiographic voltage criteria is moderate [24], but the major and minor criteria of the ST-T segment still pose reproducibility problems. In our experience, when two ECG are conducted on the same patient, in the best case scenario, the kappa index between both ECGs in relation to major and minor abnormalities is only 0.65 [95% CI 0:26 to 1:04] and 0.57 [95% CI, 0.40-0.74], respectively [25]. Our results are based solely on the performance of one single ECG. In addition, the low number of ST-T non-specific electrocardiographic abnormalities could be another limitation of our study.

On the other hand, the high rate of follow-up losses −77 patients (26%)- may have affected the results by adding a selection bias. This is another potential study limitation.

Moreover, we have no information about changes in antihypertensive drug treatment during the follow-up time. Despite these limitations, the classical cardiovascular risk factors and LVH voltage criteria maintain their prognostic value.

## Conclusions

We conclude that LVH electrocardiographic changes seen on the ECG are significantly associated with cardiovascular morbidity and mortality. However, globally non-specific repolarization disorders have no prognostic value in our cohort of hypertensive patients.

## Abbreviations

ECAP: Catalan electronic medical record system
ECG: Electrocardiogram
GP: General practitioner
HR: Hazard ratio
LVH: Left ventricular hypertrophy
MC: Minnesota Code
